# Complications of Macular Peeling

**DOI:** 10.1155/2015/467814

**Published:** 2015-09-03

**Authors:** Mónica Asencio-Duran, Beatriz Manzano-Muñoz, José Luis Vallejo-García, Jesús García-Martínez

**Affiliations:** ^1^Department of Ophthalmology, La Paz Hospital, 28046 Madrid, Spain; ^2^Department of Ophthalmology, Humanitas Clinical and Research Center, Rozzano, 20089 Milan, Italy

## Abstract

Macular peeling refers to the surgical technique for the removal of preretinal tissue or the internal limiting membrane (ILM) in the macula for several retinal disorders, ranging from epiretinal membranes (primary or secondary to diabetic retinopathy, retinal detachment…) to full-thickness macular holes, macular edema, foveal retinoschisis, and others. The technique has evolved in the last two decades, and the different instrumentations and adjuncts have progressively advanced turning into a safer, easier, and more useful tool for the vitreoretinal surgeon. Here, we describe the main milestones of macular peeling, drawing attention to its associated complications.

## 1. Introduction

Macular peeling generally refers to the surgical technique for the correction of a hole or epiretinal membrane (ERM) in the macula, or other reasons that involve the removal of the internal limiting membrane (ILM) in the central retina. Removal of preretinal macular fibrosis [1] begun shortly after the development of closed pars plana vitreoretinal surgery by Machemer and colleagues [2]. Then, bimanual surgical techniques, first used in eyes with complicated proliferative diabetic retinopathy and retinal detachments [3, 4], also permitted the resection of abnormal glial tissue from the superficial retina. The first to remove localized epiretinal membranes that were covering or distorting the macula in the absence of other primary conditions was Machemer [5], but it was popularized by many authors, who named differently such anomalies as epimacular proliferation or macular pucker [6–8].

**Figure 1:**
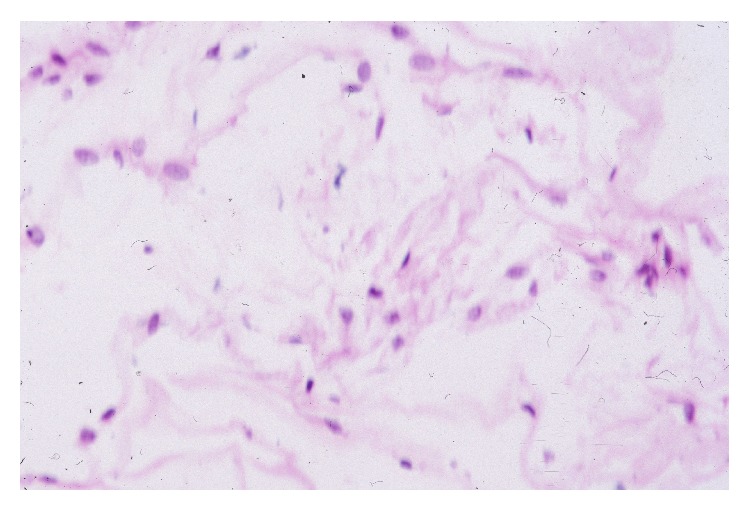
Hematoxylin and eosin 40x showing a paucicellular basement membrane composed of collagen fibers, glycosaminoglycans, laminin, fibronectin, and some astrocytes.

The ILM was first named by Pacini in 1845 and represents the boundary between the retina and the vitreous body [9]. It is a periodic acid Schiff- (PAS-) positive basement membrane, formed by astrocytes and the end feet of Müller cells and composed of collagen fibers, glycosaminoglycans, laminin, and fibronectin [10] (Figure 1).

The close association between ILM and the Müller cells suggests that it derives from these cells [10]. The macula, the parafoveal, and peripapillary regions of ILM are the thickest, measuring an average of 2.5 *μ*m in thickness, and progressively thinning to 0.5 *μ*m at the vitreous base [11].

Histological and clinical studies demonstrated that the ILM acts as a scaffold for cellular proliferation of Müller cells, thus allowing the survival of ganglion cells [12], and for migration of glial cells, creating a tangential contractile force on the macular surface and posterior vitreous cortex, sometimes with subclinical manifestations, only detected by OCT, and others leading to the formation of tight, thickened, refringent premacular posterior membranes [13]. It seems that ILM may have its main function only during early embryogenesis, and its removal would not have negative effects in the aged human eye [12].

## 2. Surgical Techniques, Instrumentations, and Adjuncts

The ILM was not clinically relevant until surgical removal of ERM by means of vitrectomy in the 80's identified small fragments of ILM adherent to the surgical specimens [14]. Posteriorly, a technique for repairing sub-ILM macular hemorrhages in patients with Terson syndrome with intentional ILM extraction was presented at the Annual Meeting of the American Academy of Ophthalmology in 1990, with excellent results which guided the authors to consider the technique of ILM removal in all forms of tractional maculopathy [15]. After the 90's, the technique became widely extended until accepted today because not only it releases these contractile forces, but also it guarantees complete separation of the posterior hyaloid from the macular surface [16] and also decreases the risk of postoperative ERM formation [17].

The ILM peeling can be achieved after a standard pars plana vitrectomy (PPV), in which a careful detachment and remotion of the posterior hyaloid intend to avoid any possible scaffolding for cellular proliferation and subsequent retinal traction [18]. To detach the posterior hyaloid, there are 2 widespread maneuvers [19]: (1) by means of suction close to the optic disk with the help of the extrusion needle or vitrector tip and control pressure of up to 150 mmHg or (2) by means of mechanical elevation of the posterior hyaloid with a membrane pick or microvitreoretinal blade (MVR). Then, the macular peeling can be performed: the first step is to create an initial flap in the ILM with a sharp instrument such as pick forceps, bent MVR, or vitreoretinal forceps. Once the flap is created, the desired area of ILM is removed with the vitreoretinal forceps using circular movements around the fovea similar to a capsulorhexis and in parallel to the retinal surface [13]. The extent of ILM to be peeled varies from approximately 1 disk area centered at the fovea [20, 21] to an area extending from the superotemporal to the inferotemporal vascular arcades [22–24]. The confirmation of tissue removed during surgery can be obtained with histopathologic studies [25], but postoperatively it is clinically difficult to ascertain the area of retina denuded. Monochromatic images, obtained using the scanning laser ophthalmoscope (SLO) at wavelengths of 488 and 514 nm, were superior to color and red-free photographs [26].

## 3. Vital Dyes in Macular Surgery

Because the ILM is thin and transparent, surgical remotion can become technically challenging even for experienced surgeons especially in difficult cases such as myopic macular hole or foveoschisis. Staining of the ILM with adjuvant dyes can make the procedure easier to perform and more effective, reducing also the operating time and the mechanical trauma to the retina [27].

Several authors have reported the use of indocyanine green (ICG) and trypan blue for assisted ILM peeling in macular hole (MH) surgery, but concerns soon appeared when evidence from several clinical reports and in vitro toxicity showed worse visual outcomes with both dyes [28, 29].

## 4. ICG

ICG has been widely used since 1970 for choroidal angiography [30]. Then, anterior segment surgeons described techniques for the intraocular use of ICG dye to facilitate visualization of the endothelial cells [31] and the anterior capsule of the crystalline lens [32], but it was popularized in the very beginning of our century in several reports for vitreoretinal surgery [33–38]. ICG is highly soluble in water but poorly soluble in saline solution and must be diluted in water or in glucose 5% to prevent later precipitation in the balanced salt solution of the eye. The concentration, volume infusion, exposure time, and osmolarity of the final solution used both for ILM and ERM removal have been varied between different authors, and the staining can be done in a fully filled eye with the infusion stopped [28, 34, 35, 39–48], or after complete fluid-air exchange [41, 42]. In order to minimize the side effects of ICG on the retina, several techniques have been emerging: (1) to inject small amounts and concentrations with the infusion on and immediate suction of remains to wash the dye out rapidly [49, 50], (2) to bind remnants by placing autologous serum [51] over the area of retina lacking ILM, knowing that ICG has high affinity to lipoproteins, and (3) to prevent the access of ICG into the subretinal space in eyes with MH by using a drop of perfluorocarbon liquid [52, 53] or viscoelastic materials [54, 55]. Though several reports showed favourable anatomical and visual results with the use of ICG staining, the latest suspicions on its safety forced surgeons to compare functional outcomes with and without the use of the dye. In this respect, numerous studies reported poorer visual results when ICG was used to stain both ILM and ERM [44, 56, 57]. In spite of the fact that ICG could induce a rigidness and detachment of the ILM and facilitate its removal with staining, Gandorfer and Haritoglou found on histopathologic studies fragments of Müller cells and other undetermined retinal structures adherent to the retinal side of the ILM, suggesting that intravitreal application of ICG may cause retinal damage by altering the cleavage plane to the innermost retinal layers [28, 58, 59]. These structural findings were confirmed in a donor human eye [60] and in nonhuman eyes [61].

Others suggested unusual atrophic changes in the retinal pigment epithelium (RPE) on the site of the previous macular hole or in the area where the ICG solution would have had direct access to the bare RPE cells [39, 47, 62]. In experimental models, it was demonstrated that subretinal delivery of ICG was able to induce as much RPE as photoreceptor and outer nuclear layer damage [63, 64], especially if the eye was air filled [65]. It has been hypothesized that the RPE damage could be related to direct toxicity of the dye to these cells [66]. Some authors attributed an enhanced toxic effect of ICG staining with intense light exposure [67, 68], so that Ho and colleagues proposed to remove the sodium from the solvent for the dye preparation in order to reduce the cytotoxicity [69].

Phototoxicity alone has been studied as a possible cause for RPE cells damage induced by ICG, due to its absorption spectrum (700–800 nm) in front of the emission spectrum of current light sources employed in PPV (380–760 nm) [70, 71]. This deleterious effect could be reduced through the intake of 10 mg/day of oral lutein several days before surgery, according to Wu and colleagues [66].

There also have been described visual field defects with the use of ICG staining: from small nasal scotomas to nasal hemianopsia, whose mechanism of production is not yet well understood [28, 56, 72, 73]. Slimming of the retinal nerve fiber layer [74] or damage to the retinal ganglion cells (RGC) with high concentrations of ICG has been hypothesized [75]. Persistence of the dye seen as fluorescence at the optic disk has been detected in eyes in which ICG was employed up to 2 years after the macular surgery [76–80]; this finding could be related to an uptake of ICG by RPE cells through the hole in cases of MH [77]. Other authors have detected a reduction of the b-wave in experimental electroretinograms after the exposure to ICG, suggesting some degree of inner retinal damage [81].

In spite of the fact that there are many reports suggesting the possibility of ICG toxicity to the retina and RPE, experimental toxicity may not correlate exactly with actual clinical application of ICG, in which the intraoperative conditions can be much different. There is notable laboratory experimentation to the contrary demonstrating that even at high concentrations followed by maximum power illumination for 3 minutes ICG caused no histologically detectable damage [82]. Taking into account the differences in species and in vivo-ex vivo studies, this raises the possibility that either the ICG instillation or the infusion [83] or fluid-air exchange [84, 85] might have hydrodissected the ILM from the underlying retina and injured the retinal nerve fiber layer. Indeed, if ICG instillation hydrodissected the ILM from the retina, the ICG solution would have had direct access to the retinal tissue, which might help to explain their reported photodynamic effects [86].

## 5. Infracyanine Green

Infracyanine green (IFCG), unlike ICG, does not contain iodine, and it needs glucose 5% to solve in water, but it is isoosmolar, which would reduce the potential for retinal toxicity, compared to ICG [87], and hypoosmolar related to vitreous humor. IFCG has been used to stain both ILM and ERM without serious clinical adverse events [88–92]. In histopathologic studies of ILM specimens obtained from MH and diabetic macular edema (DME) eyes, 80% contained remnants of Müller cells footplates, neural cells, and ganglion cells [93, 94], suggesting would create the same cleavage plane of the ILM as ICG. Nevertheless, no evidence of acute or delayed permanent damage to the RPE at different concentrations of IFCG or in combination with endoillumination was found [95]. In spite of its apparent safety, its use is not very widespread.

## 6. Trypan Blue

Trypan blue (TB) has been widely used in anterior segment surgery to stain corneal endothelial cells [96] and lens capsule [97]. In vitreoretinal surgery, it has been used to stain the posterior hyaloid, the ERM, and the ILM [98–105]. The mixture with glucose 10% allows adequate staining for both ERM and ILM without detectable toxic side effects [98–101, 103–108], but some authors established that, due to the cellular affinity of TB, the dye would not stain properly the acellular ILM [109] and would need to be used under air for a longer time to improve staining of ILM [110]. Reports comparing TB with ICG found better visual outcomes with trypan blue assisted ILM peeling [111], and no clinical [107, 112] or experimental acute damage was observed [108, 113], although some authors detected some retinal disorganization at concentrations of more than 0.15% [81, 108, 114], or exposure times of more than 2 minutes [115].

## 7. Other Dyes

Triamcinolone acetonide (TA) was first used by Tano intravitreally in 1980 [116], after being used in ophthalmology to treat many ophthalmic diseases. This water-insoluble steroid aids in the visualization of vitreous, upon the insoluble nature of the white crystals and the integration into loosely organized collagen matrices [117–120]. An extension of this mechanism is thought to be responsible for the staining of the superficial portions of an ILM [121–127] and ERM [120]. The drug is commercially available in an aqueous suspension and has been administered with or without the removal of its solvent in the second case in order to avoid possible toxic effects [117, 120, 122, 123, 127]. Different methods such as sedimentation or filtration techniques and centrifugation [128] are usually used to eliminate the solvent, usually benzyl alcohol, from the preparations. Lately, new products based on TA have appeared that can be injected directly into the eye. Other possible adverse events of TA are increase in intraocular pressure [122, 124, 125, 127, 129, 130], generally transient and controlled medically, cataract progression [129, 130], or, in some cases, endophthalmitis that has been described as infectious (more delayed and painless than usual) or noninfectious (more acute, in which hypopyon may represent the TA material itself or a sterile inflammatory reaction) [131].

Brilliant Blue G (BBG), also known as acid blue 90 or Coomassie BBG, was first reported in vitreoretinal surgery by Enaida et al. and has been used specifically for the staining of the ILM [132] with good morphological and functional results [133–135]. The dye stains badly the ERM, but some authors performed double BBG staining and double peeling for both ERM and ILM in order to prevent ERM recurrence [136]. Recently, a mixture of TB and BBG solution for staining both the ERM and ILM simultaneously avoided the need for fluid-air exchange [137, 138].

Patent blue is another blue dye which was first used in cataract surgery for anterior lens capsule staining [81]. It has been used posteriorly for both ERM and ILM removal with mild staining [139] and without clinical adverse events at 6-month follow-up in small series [140], although more studies are needed to evaluate the efficacy and the safety of the drug. Novel promising vital dyes are under investigation in an in vitro and in vivo models that may be useful for vitreoretinal surgery like lutein and zeaxanthin-based natural solutions.

## 8. Indications for Macular Peeling

In idiopathic MH, ILM peeling relieves foveal traction from the retinal surface [141–143] by complete removal of any epiretinal tissues and by stimulation of gliosis [61], therefore shortening the face-down period in the post-op and the need for the use of long-acting gas [144–146], with better anatomical closure rates but not better visual improvement [147]. In myopic FTMH, the mechanism of hole formation is more complex and involves not only tangential and/or anteroposterior traction, some authors suggested that the ILM could have a role in the development of foveal retinoschisis that frequently accompanies these cases [148]. Several reports support this reasoning with better visual results and higher definitive closure rates when ILM peeling was performed [149–151]. When FTMH is secondary to trauma and does not resolve by itself (which occurs in up to 44.4% [152]), PPV with removal of posterior hyaloid, ILM peeling, and gas tamponade can obtain the best anatomic success over other techniques [23, 153].

Epiretinal membranes began to be routinely removed by PPV from 1978 [14]. Surgery is recommended in both idiopathic and secondary membranes in eyes whose vision is significantly reduced by the ERM, although secondary ERMs showed a greater amount of improvement than idiopathic ones [154, 155]. Also, as ILM may act as a scaffold for reproliferation, ILM peeling can not only prevent a recurrent postoperative formation of ERM [37, 147, 156–158] but also reduce the preoperative cystoid macular edema associated with ERMs [159].

ILM peeling has been used in some cases of refractory diabetic macular edema (DME) after failed intravitreal injections of anti-VEGF, steroids, and/or laser photocoagulation, with decrease in foveal thickness but with no improvement of visual acuity postoperatively [160–163]. In branch and central retinal vein occlusion-associated macular edema, there are few series of selected cases that show improvement in visual acuity after PPV with the removal of preretinal hyaloid and peeling of the ILM [164–166]. As in DME, PPV alone can provide better retinal oxygenation [167], but ILM peeling could help in pumping blood and fluid from the retina into the vitreous cavity [164] and could also reduce the recurrence rate of both macular edema and ERM compared to PPV alone [168–171].

Other possible applications of macular peeling are optic disk maculopathy [172], vitreomacular traction syndrome [173], Terson's syndrome [174], and prevention of ERM formation in retinal detachment surgery [175, 176]. Dithmar also reports a case of soft confluent drusen absorption after ILM peeling [177].

## 9. Complications of Macular Peeling

There are some complications after macular peeling that are common to other vitreoretinal procedures, probably more related to PPV than peeling maneuvers, even in the era of microincision surgery [178, 179], like cataract progression [86, 147, 180–182], intraocular pressure increase [46, 182–184], visual fields defects [28, 181, 185–187], retinal tears [22, 86, 151, 182, 188–190], retinal detachment [46, 73, 86, 150, 151, 181, 188, 191–193], vitreous hemorrhage [46, 194], ocular hypotony [195], dislocation of the intraocular lens in pseudophakic eyes [86, 192], macular phototoxicity [188], RPE changes [20, 39, 193], and endophthalmitis [191, 196, 197].

There are other complications directly attributable to macular peeling, including focal retinal hemorrhages and edema, which generally resolves spontaneously without the need of treatment [20, 23, 188, 198]. Paracentral scotomas and visual field defects, usually asymptomatic, have also been reported but not directly correlated with the removal of the ILM and could result from adjuvant stain or mechanical trauma to the nerve fiber layer (RNFL) [20, 44, 74, 198–200]. There are also few reports about retinoschisis [199] and macular edema after macular peeling [20, 201]. Karacorlu described small punctate lesions of the RPE and choriocapillaris attributed to ILM grasping during the surgery that do not appear to affect the surgical outcome [202].

The earliest change in the macula is postoperative swelling of the arcuate RNFL, which disappears within 3 months. It appears as hypoautofluorescent arcuate striae in the macular region on infrared and autofluorescence imaging, with corresponding hyperreflectant swelling demonstrated on spectral-domain optical coherence tomography (OCT) [200]. This is followed by dissociated optic nerve fiber layer (DONFL), now detectable on fundus examination with blue filters in half of the eyes, as arcuate dark striae along the course of the RNFL [203, 204], or as concentric macular dark spots on the en-face OCT [205]. The correspondent image on OCT is seen as “dimples” in the inner retinal layers that seem to be the result of an interplay between trauma and healing processes constrained by nerve fiber layer [206] and it is not associated with adverse effects on the visual function, as detected by visual acuity and scanning laser ophthalmoscopy microperimetry [203, 204, 207, 208]. Postoperative foveal displacement toward the optic disc has been also described after both ERM and ILM peeling [209, 210] and it might be responsible for the stretching and thinning of the retinal parenchyma in the temporal subfield with the thickening of the nasal macula. This is probably secondary to axonal transport and contractility alterations in the RNFL, due to apoptotic and atrophic degeneration on the peripapillary area [200]. Ganglion cells do not seem to be affected by ILM peeling, although some authors detected a reduction in the inner plexiform layer thickness by OCT imaging at 6 months after BBG-assisted surgery, because of trauma to the Müller cells contained in the ganglion cell layer [211]. It is not consistent with other retrospective study that found up to 46.7% of optic nerve atrophy 6 months after ICG-assisted surgery, which caused irreversible peripheral nasal visual field defect, so that would need longer follow-up investigation [72].

**Figure 2:**
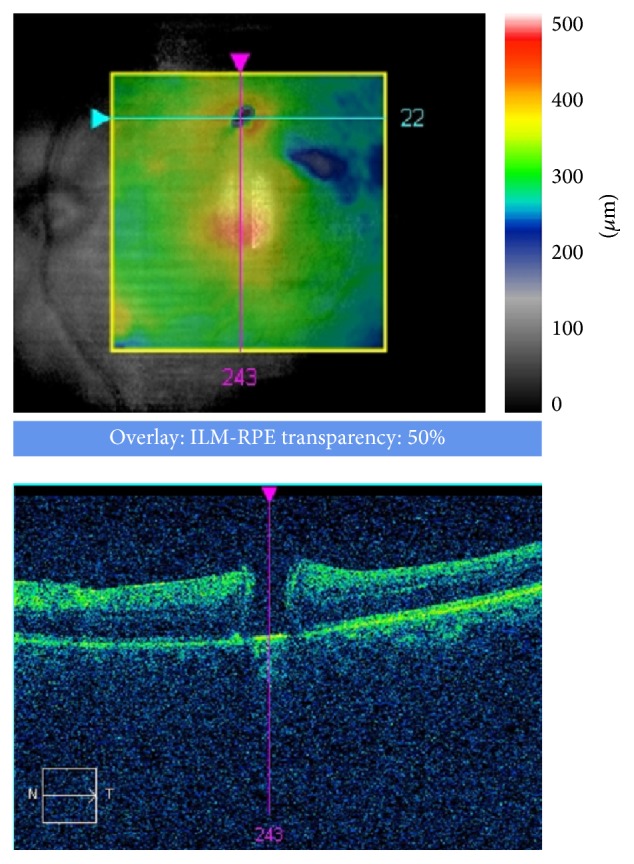
Parafoveal iatrogenic macular hole 1 week after ILM peeling for a full-thickness macular hole (FTMH).

Iatrogenic eccentric full-thickness retinal breaks have been documented after ERM and ILM removal in idiopathic FTMH and DME [204–207], with an average incidence of 0.6% [212]. Usually, they present bright fluorescence on autofluorescence imaging and as flat full-thickness holes on OCT (Figure 2).

Sandali and colleagues. did not found iatrogenic macular holes or choroidal neovascularization in any of the retrospective series of 909 patients with a mean follow-up of two years, but proximity to the fovea correlated well with a worse visual prognosis [212]. It is believed that the location of the holes represents the initial or the end site of ILM elevation, or the result of a weakening in the glial structure of the retina [90, 212, 213]. Some authors propose a modification of the peeling avoiding the foveolar ILM in order to prevent retinal inner changes and probably achieving better final visual outcomes [214].

There are some reports of retained intraretinal emulsified silicone oil and gas bubble after ILM removal and endotamponade with these agents that contributed to the surgery failure [215, 216].

Microtrauma to the RPE and defects in Bruch's membrane are thought to be the origin of rare complications reported like choroidal neovascularization or formation of RAP-like lesions [217–219], and it seems that prior age or trauma-related changes and surgical trauma are predisposing factors for its development.

Uemoto described 2 cases of an epimacular proliferative response after ILM peeling, related to the injury but not progressing after 2 years [143].

Subretinal hemorrhage and subsequent vitreous hemorrhage are other complications that can occur after ILM removal for FTMH [220]. The latter can occur even in the absence of retinal hemorrhage in hypertensive patients [221].

## 10. Discussion

Comparing series with and without ILM peeling, all but one study [14] reported statistically significant improved outcomes if the ILM was peeled. Internal limiting membrane removal appears to be especially beneficial in eyes with primary surgical failure or reopened/large/chronic holes [14]. A literature meta-analysis, reviewing 31 studies involving 1,654 eyes undergoing macular hole surgery, compared three different surgical techniques: no adjuvant, no ILM peeling; adjuvant, no ILM peeling; and no adjuvant, ILM peeling. There was no statistically significant difference between the first two methods, but ILM removal resulted in statistically significant (*P* < 0.0001) better anatomical and functional outcomes over both the other techniques [222]. In a prospective multicenter randomized controlled trial with 141 patients, although there was no evidence of a better distance visual acuity after the ILM peeling versus no ILM peeling techniques, a benefit in favor of no ILM peeling was ruled out, but it seemed to be the treatment of choice for idiopathic stages 2 to 3 FTMH [223]. It must be taken into account that ILM peeling can be a traumatic procedure that has acute adverse effects on the underlying retinal layers and even in the RPE and choriocapillaris. Further investigation of these subclinical changes may assist in aiding the development and improvement of minimally traumatic techniques for ILM removal.

## 11. Conclusions

The combined ERM-ILM peeling for the correction of macular ERM and the ILM peeling for the correction of MH and its variations are useful techniques in the new era of microvitreoretinal sugery, usually with good anatomical and functional outcomes, but they can have a little proportion of complications (toxic or mechanical, transient, or irreversible), even in hands of experienced surgeons, which must be taken into consideration in order to achieve the best results.
